# Crito, in Answer to a Derbyshire Practitioner

**Published:** 1807-10

**Authors:** 


### To the Editors of the Medical and Physical Journal.

Gentlemen,

YOUR Readers, who are pleased to pay attention to Dr. Cayley's Case of Aneurism, in your 96th Nnmber, and my Observations upon it in your 99th, will soon perceive that the inference I produce is from his own words, and agreeable to truth. The case was certainly of a doubtful nature, otherwise Mr. Strother could have been at no loss in distinguishing it; neither could I have been induced to take any notice of it, had not Dr. Cayley's *candour* to Dr. Stubbs been so very conspicuous. I am neither acquainted with Dr. Cayley nor Dr. Stubbs, so that I can bear no ill will to the one, or favour for the other. Justice requires impartiality, which ought always to be the principal object both in our writings and transactions with mankind. Dr. Stubbs has not come forward himself, to vindicate his character, or give us an account of the case, and the reasons which induced him to propose caustic. This may be owing, perhaps, to his ignorance of any attack being made against him. There are ten medical men in our town, and only four copies of your useful Journal come to it; of course, the other six might remain ignorant of any thing said against their practice, if some of their friends did not acquaint them with it. Dr. Cayley, on the other hand, has not given us an impartial account of this character, whose plan of treatment must have been related to him by second tongue, which seldom repeats it like the first. Were Dr. Stubbs to favour us with his account of the affair, I suppose it would be very different. " This is the ground on which I have undertaken his defence, from ignorance and illiberality. We often find, that Doctors do not always use honourable language to every one regularly educated, and honourably pursuing his profession. Allowing Dr. Stubbs to be an empiric; the language used, had more the appearance of coming from a Dictator than from a meek physician.

We are sorry, says the Derbyshire Practitioner, that Mr. Strother should have been so unnecessarily brought forward. Was he not first called? Did he not heal the wound, which was inflicted by the point of a sheers, by the first intention? Did he not poultice the aneurism for a *long* time? (the length of time is not mentioned.) Was he not going to open it with a lancet, considering it as an abscess? I allow with the practitioner, that every purpose might might have been answered, without either the names or modes of treatment of Messrs. Strother and Stubbs being mentioned: but the case must have assumed another dress. He then proceeds, " Indeed, at present he has committed no material error; on the contrary, he *has* corrected his first opinion, and *has* discovered and anounced the disease before Dr. Cayley's presence was required.

I believe no one would think, who knows any thing about the arterial system, of applying caustic to aneurism seated upon any of the large trunks, where the patient is exposed to imminent danger, even when the operation is performed by the most expert surgeon. But in a case, like that of Dr. Cayley's, I would have ventured its application without endangering the patient's life; and when he was not willing to undergo the operation, should have proposed it as the next practical method: but in all cases, where it is necessary, I would certainly prefer the operation as most expeditious, and certain. I still have my doubts whether this was true aneurism, several varieties of which are mentioned by authors — The encysted—diffused—and varicose, each of which requires a different mode of treatment. Compression has been recommended by almost every writer upon surgery, with various success. Sometimes it has been found to do good, and at other times mischief. A case of the diffused kind is recorded by Dionis, where four pounds of blood were taken from the superior part of the arm, after the operation of aneurism; where it had been lodged by previous compression. The contents of encysted aneurism, by a very gentle compression, are sometimes made to disappear; it is in such a case as this where compression could have any chance of succeeding; here, aneurism might be considered as an arterial hernia, and could compression be applied without stopping the circulation in the veins or straining the valves, it ought to be tied. An aneurismal truss might be easily contrived for the purpose. Dr. Adams has used something of this kind for compressing the artery in a species of aneurism, which terminated favourably. (See Number 34.) It will shew the wonderful power of Nature in this complaint. Whether the artery was really obliterated by the pressure, Dr. Adams has his doubts, but a very useful fact is afforded us, from which we may derive advantage, not only here, but in every other case of wounded artery; that the blood may be coagulated in them by pressure, and by this means their diameters may be contracted; " and as the pain during pressure arose from the artery overcoming that pressure, it is obvious that the remedy is to increase it; and the success I met with justifies such a conclusion."

Dr. Hunter has considerably enlarged our knowledge of the treatment of varicose aneurism; he informs us, that the operation of obliterating the cavity of the artery, is seldom or ever necessary; that the aneurismal tumour does not here proceed so rapidly; and that when it increases to a certain size, it remains stationary without acquiring additional bulk, and that it may subsist for many years without being productive of much inconvenience to the patient. This is a very important circumstance, as it saves the artery, and the trouble of a very painful operation.

When aneurism is seated upon a part where the operation cannot be performed, gentle compression, blood-letting, a low diet, abstinence from exercise, and the use of opium when indicated by pain, are the only remedies to be depended upon. I believe there are very few instances of Nature curing an aneurism, or gradually obliterating the artery, without the assistance of art. Yet after the operation for aneurism, when it has been suspected that the main trunk has been obliterated, pulsation has been felt at the wrist and ancle. There is no doubt but the branches which were before only secondary, become of more importance, and supply the parts with a greater proportion of blood. On the other hand, instances have been known, where, instead of a return of circulation and sensation, the extremities have become dead, cold, and insensible! And from the want of circulation, the ultimate stage of gangrene has been the consequence. Having pointed out some of the leading particulars in the treatment of this disease, I shall proceed to comment upon the remarks of the Derbyshire Practitioner. He observes, "Dr. Bailey is mistaken in another point." In what? "That the blood was very readily forced into the artery by gradual pressure." Of this circumstance Dr. Cayley must have been a better judge than him. The disappearance of the tumour upon pressure, is one of the diagnostics of the encysted aneurism. In the diffused, the blood will diffuse of itself without much compression, and by this means it can be easily distinguished from the encysted; which of the species of aneurism does the Derbyshire Practitioner think this case to have been? Throughout the whole remarks, he perverts my meaning as well as Dr. Cayley's. He says, I attribute every thing bad to compression. Where does he find me say that He seems, however, to condemn condemn every kind of compression, and says, "In the general run of practice it is not likely to be much followed. This point, in surgery, seems to be so well settled, as to require but little argument now to enforce it." The Derbyshire Practitioner cannot positively say that it was the anterior tibial artery that was wounded, because we are not told upon what part of the leg the wound was inflicted. A copious hæmorrhage attended, but this was restrained by the pressure of the thumb only, till Mr. Strother was brought from Pately, a distance of three miles, and this hæmorrhage did not afterwards return. They stopped the hæmorrhage themselves, and when Mr. Strother came, all was over; he certainly had nothing to do but bind up the wound. Dr. Cayley does not say that Mr. Strother boldly advanced his finger into the wound to encounter at once the bleeding orifice. The Practitioner has his imagination very much upon the tiptoe in this place. We have no account of the manner in which Mr. Strother treated the wound when he first visited the patient; a blank which ought to be filled.

"To lessen the importance of the arteries of the leg," I am charged with calling them branches of the popliteal artery. The Derbyshire Practitioner will not allow the tibials to be branches of the popliteal; but every anatomist describes them as such; calling them, therefore, what they are, is not lessening their importance. "The crural artery descends on the inner part of the thigh, close to the os femoris, sending off branches to the muscles, and then sinking deeper in the hind part of the thigh, reaches the ham, where it takes the name of *popliteal*: after this it separates into two considerable *branches*, one of which is called the anterior tibial artery; the other divides into two branches; and these arteries all go to be distributed to the leg and foot."

He next proceeds to my case of wounded ulnar artery. I do not wish any one to think that there is any superior intelligence manifested in it. I am only sorry that he does not understand what I meant by sewing the artery. There are two methods of securing an artery; the one is by the tenaculum, and the other by the curved or crooked needle. The tenaculum is certainly preferable; but sometimes when the wound is deep, or the artery buried so that it cannot be laid hold of with the tenaculum, the needle must then be used. Owing to the swelling upon the part, it was found impossible to secure the artery by the tenaculum; securing it with the needle was then had recourse to; I hope this will satisfy him with respect to the term which

I have used. But it has occurred to me, that perhaps he might have thought I had sewed the artery in the manner proposed by Mr. Lambert of Newcastle. He recommends the twisted suture. The Practitioner must think that I have a very nice eye, and great dexterity of hand, if I could give it the glover's stitch. Several objections might be urged against Mr. Lambert's ingenious proposal of sewing the artery in the operation for aneurism; but I shall not enter upon them at present, nor the Practitioner's query about "The nice opposition of the edges of the wound in an artery *affecting its calibre,*" which was one of the objections urged against Mr. L's proposal. It is very well that the Practitioner agrees with me about the impropriety of the term *cicatrization,* as used by Dr. Cayley. The internal surface of an artery must be very different from what I have any conception of, if by compression their sides could be cicatrized. The Practitioner is for obliterating the artery at *once* by a single pull of the ligature.— This will do very well upon paper; bnt cases will happen where one single pull of the ligature is not practicable, as in the case I related, where the man's arm was swelled to the greatness of his thigh.

A similar case, where the ulnar artery was cut with a knife, occurred here sometime previous to the one I have related, which terminated fatally. I did not see it myself, but I was informed that great œdematous swelling and gangrene came on, which soon put a period to the patient's existence Men of professional consequence attended the case; but had the Derbyshire Practitioner been called, he would have obliterated the artery by a single pull. I shall not transgress farther upon the patience of your readers. I have already observed, that caustic is highly improper to be applied to aneurismal tumour seated upon the large arterial trunks. In other cases, I believe it might be used without endangering the patient's life, which was all that I meant to infer. I am glad that I have not *discomposed* your mind; as to illuminating it, I shall leave for an opportunity of leisure; which is not the case at present. I do not know what might be the consequence, if the encysted aneurism upon any of the large arteries were to be punctured by one of Mr. Hey's needles, nor do I wish to detract from their usefulness in exploring the contents of tumours of a doubtful nature. It is, however, a very happy circumstance, that few surgeons can be at a loss in distinguishing aneursim.

*July,* 20, 1807. CRITO.

